# Domestic violence shapes Colombian women’s partner choices

**DOI:** 10.1007/s00265-017-2405-2

**Published:** 2017-11-19

**Authors:** Martha Lucia Borras-Guevara, Carlota Batres, David I. Perrett

**Affiliations:** 1 Perception Lab, School of Psychology and Neuroscience, St Mary’s Quad, University of St Andrews, St Andrews, Fife, Scotland KY16 9JP UK; 2 Present Address: Psychology Department, Gettysburg College, 300 North Washington Street, Gettysburg, PA 17325 USA

**Keywords:** Masculinity, Public violence, Domestic violence, Partner preferences, Intra-sexual selection

## Abstract

**Abstract:**

Potential protection from violence has been suggested as an explanation for women’s preferences for more masculine partners. Previous studies, however, have not considered that violence may be multi-modal, and hence come from different sources. Therefore, we tested the effect of different fears of violence (i.e. vulnerability to public crime, likelihood of within-partnership violence) on masculinity preferences of women from Colombia, a country known for its high rates of violence. Eighty-three adult heterosexual women (mean age ± SD = 26.7 ± 6.01) answered a survey that included questions about health (e.g. frequency of illnesses during the last year and during childhood), access to media (e.g. time spent watching television, frequency of internet use), education (i.e. highest level achieved) and violence perceptions. Participants’ masculinity preferences for Salvadoran, European and Colombian male faces were recorded. Factor analysis revealed two different factors for the answers to questions related to violence. One factor loaded mostly on questions related to public violence and the second factor related to domestic violence. We found that women with higher scores on the domestic violence factor preferred significantly less masculine Colombian male faces. Even after controlling for participant age, education, access to media (TV and internet) and health-related factors, the domestic violence factor contributed significantly to explaining masculinity preferences. The results presented here suggest that women’s preferences for masculinity may be a strategy to avoid aggressive partners and that the source of violence matters in mate choice.

**Significance statement:**

Women who perceive higher risks of domestic violence prefer less masculine looking partners. Using an experimental approach, we show that Colombian women who feel more in danger of violence within partnership prefer the faces of less masculine males. This was true even after controlling for women’s education level, health and access to media.

**Electronic supplementary material:**

The online version of this article (10.1007/s00265-017-2405-2) contains supplementary material, which is available to authorized users.

## Introduction

Masculine male traits, across different taxa, are associated with genetic and physical traits which could be beneficial during sexual selection (Emlen 2008; Santos et al. 2011). These traits include, but are not limited to, fighting ability (Bergeron et al. 2010), higher dominance rank (Marty et al. 2009), physical strength (Malo et al. 2009) and fertility (Preston et al. 2003). In humans, the same pattern exists, with masculinity correlating positively with quality (e.g. reproductive success; Apicella et al. 2007), physical traits (i.e. sexual dimorphism; Thornhill and Gangestad 2006) and dominance (Batres et al. 2015) but also with negative personality traits (e.g. infidelity; O’Connor et al. 2012).

Female mate selection and/or male-male competition are possible driving forces behind preferences for masculinity (Barber 1995; Puts 2009; Little et al. 2011a; Puts et al. 2012). Most research has focused on the role of female mate selection, both at the individual and population level (Rhodes et al. 2003; Thornhill and Gangestad 2006; DeBruine et al. 2011; Rantala et al. 2013; Batres and Perrett 2014), whereas little attention has been given to the influence of male-male competition (Snyder et al. 2011; Batres and Perrett 2014; Batres et al. 2015).

At the individual level (measures that vary within a population), masculine men report an increased preference for casual relationships (Rhodes et al. 2005; Boothroyd et al. 2008, 2011), being more aggressive (Puts et al. 2012) and are perceived by women as more likely of being unfaithful (O’Connor et al. 2012) than their feminine counterparts. In some cases, men’s masculinity has been argued to be an honest indicator of health. For example, Thornhill and Gangestad (2006) found that self-reported frequency and duration of respiratory diseases negatively correlated with men’s masculinity. Likewise, masculinity positively correlated with medical records of health and immune function response to a vaccine (Rhodes et al. 2003; Rantala et al. 2012; but see Rantala et al. 2013). Nonetheless, measures of genetic quality (e.g. major histocompatibility complex diversity) were found not to predict facial masculinity in men (Lie, Rhodes and Simmons 2008; see Scott et al. 2013 for a review). In terms of physical traits such as strength (Fink et al. 2007) and formidability (Wolff and Puts 2010), these correlated positively with facial ratings of masculinity in men. Snyder et al. (2011) found that women who felt more vulnerable to crime preferred more formidable male partners, suggesting that women prefer masculine partners in environments where protection is needed (Snyder et al. 2011). All considered, studies at the individual level suggest that masculinity provides a clear cue to men’s personality and physicality but it is less clear that masculinity provides an unambiguous cue to health. As a result, women choosing a masculine male partner face a dilemma between wanting a partner who is strong, formidable, perhaps healthier but less likely to commit to a long-term relationship or one who is weak, less formidable and healthy but more faithful.

At the population level, research on women’s masculinity preferences has focused on the association with aggregated indicators. Country-level measures of access to education, media (internet use frequency), health (parasite load) and violence (homicide rate, income inequality) have been studied (e.g. Brooks et al. 2010; DeBruine et al. 2010, 2011; Batres and Perrett 2014). Reduced access to education for women leads to a preference for men with higher-resource acquisition power (which has been positively correlated to masculinity, Kasser and Sharma 1999). Regarding health, women in countries or US states where health is better showed a lower facial masculinity preference for potential male partners (DeBruine et al. 2010, 2011). Likewise, Penton-Voak et al. (2004) argued that British women had a lower preference for masculine male faces than Jamaican women because Jamaica may have a higher pathogen load. These studies attributed their results to masculinity being an honest indicator of health, which would be more important to women in environments with a higher pathogen load. In contrast, Scott et al. (2014) found that women living in populations with a higher disease burden preferred less masculine male partners and that urbanisation level was a better predictor of masculinity preferences than any population health measure. Moreover, other ecological variables have been shown to influence masculinity preferences. For example, media access had a significant effect on Salvadorans masculinity preferences: people who had access to the internet preferred more masculine male faces than people who did not (Batres and Perrett 2014). Batres and Perrett (2014) suggest the media effect may arise from masculinity being portrayed in social media as an attractive physical trait.

These studies are not consistent in showing that masculinity is preferred because it signals better health. Two main possibilities could explain the discrepancies. First, male-male competition may be an important factor that has been disregarded by researchers (Puts 2009). Second, variation in masculinity preferences (Gangestad and Simpson 2000; Little et al. 2011a) could be a reflection of the trade-offs women face when choosing a masculine partner in different environments. For example, when living in infection-prone environments, women may show a higher preference for traits related to health (Tybur and Gangestad 2011; Little et al. 2011b) but, in environments where access to resources is difficult, women may prefer more feminine partners as this trait is associated with being more cooperative (Little et al. 2007).

On the other hand, when studies of women’s masculinity preferences have focused on the effects of male-male competition, the results have been consistent at different levels of analysis. At the individual level, women who felt more vulnerable to violence from strangers preferred formidable male partners (Snyder et al. 2011). Additionally, women who felt more at risk in public places preferred higher formidability and dominance in potential partners (Ryder et al. 2016). Furthermore, women who were primed with images of male-male conflict preferred more masculine male faces than women who were shown a neutral prime (Little et al. 2013; Li et al. 2014). This literature suggests that, if faced with an antagonistic encounter, men who are more masculine, formidable and dominant would be expected to be better equipped to win. At the population level, women living in countries with higher income inequality, which is associated with increased violence, showed higher preferences for masculine male partners. Women’s masculinity preferences were better predicted by income inequality than by a country’s health index (Brooks et al. 2010; but see Debruine et al. 2011). These results have been explained in terms of women preferring men who are more able to defend themselves and their partners in environments where there is high risk of conflict (Brooks et al. 2010; Puts 2010).

It must be considered that a masculine (more aggressive, dominant) partner increases a woman’s risk of being subjected to violence within the relationship as well. Women’s anger and disgust increase when shown images of men’s aggression towards women. This emotional reaction triggered women’s preferences to switch away from masculine male voices and faces in potential partners (Li et al. 2014). Hence, when there is danger of women being the target of aggressive behaviour, women prefer less masculine characteristics. This lies in contrast to the priming of increased masculinity preferences when women were are shown men’s aggression towards men (Li et al. 2014). Additionally, Colombian women who agreed with the statement “men are dangerous to their children” had low masculinity preferences for male faces (Borras-Guevara et al. 2017). Following the trade-off theory, the above literature suggests that the violence source coming either from strangers (public violence) or from partners (domestic violence), could have different effects on women’s masculinity preferences, since the types of violence could be associated with different costs and benefits. A more dominant, masculine partner may be an asset when the source of violence comes from outside the household but could surely be a liability if the violence comes from within the household.

Field studies on the effect of violence on masculinity preferences have hinted at differential effects depending on the source of violence, either from strangers or from within partnership (Borras-Guevara et al. 2017). Priming research in the laboratory has also suggested that women’s masculinity preferences are affected differently depending on the type of violence they are exposed to (Li et al. 2014). Our aim is to build from previous evidence to get to a better understanding of how women’s masculinity preferences are affected by violence. Questions related to violence used by Borras-Guevara et al. (2017) were general; hence in the current study, we ask more specific questions that relate to different types of violence. Likewise, the results of Li et al. (2014), although valuable, are limited as participants in this study were undergraduate, first world students, which means that the sample may not be representative of a general population (Henrich et al. 2010). There is a need for both experimental and field studies. In experimental studies, it is possible to isolate specific influences yet the effects of priming may last minutes and may not reflect the same processes that drive preferences outside the laboratory. In societies with more violence, effects on masculinity preferences may be more marked.

In the present study, we investigate masculinity preferences of Colombian women from both urban and rural areas and therefore a range of backgrounds. We include questions relevant to different types of violence to test empirically whether concerns of different types of violence can be subsumed into a single construct or whether they can be differentiated into distinct types of concern over public and domestic violence. If different fears are separable, then we predict that women’s masculinity preferences will be *higher* when they perceive a higher risk of public violence due to the need for protection. This first prediction arises since masculinity is associated with strength and formidability (Fink et al. 2007; Wolff and Puts 2010), traits that are desired in a partner by women who feel more at risk in public places (Snyder et al. 2011; Ryder et al. 2016). We also predict *lower* masculinity preferences when women feel a higher risk of domestic violence since masculinity has been related to men being perceived as dangerous to their children, and being more aggressive, stronger and formidable (Fink et al. 2007; Wolff and Puts, 2010; Puts et al. 2012; Borras-Guevara et al. 2017).

As previous studies have shown that the ethnicity of faces shown to participants influences face judgements, we predict that the influence of violence will be more relevant for own-ethnicity (Colombian) facial stimuli. Stephen et al. (2012) showed that attractiveness ratings were better predicted by colour with own-ethnicity faces. Likewise, Borras-Guevara et al. (2017) found that women’s masculinity preferences differed depending on the ethnicity of the face. Employing stimuli ethnically closer to participants may thus lead to more ecologically valid conclusions. Being able to compare masculinity preferences for three different face ethnicities (European, Salvadoran and Colombian) ranging in closeness to the participant population will enable us to differentiate whether preferences are associated with the physical construct of masculinity, independent of face ethnicity, or reflect a more culturally specific construct of masculinity. We define closeness in terms of ethnicity descent and geography. Colombia and El Salvador are both Hispanic countries separated by a distance of approximately 1200 km compared to a distance of 9500 km between Colombia and Europe. Concerning ancestry, Colombians are descended mostly from a mix of Europeans, Amerindians and Africans. By contrast, Salvadorans are descended mostly from Europeans and Native Americans. We therefore predict that effects of violence will be most evident for own-ethnicity faces and less apparent for other-ethnicity faces.

For the past few decades, different researchers have debated about what drives women’s masculinity preferences: either female mate choice (DeBruine et al. 2010, 2011) or male-male competition (Puts 2009; Brooks et al. 2010). We hope that the current study will help clarify this issue. Our study attempts to understand the effects of different sources of violence but since previous studies have related women’s masculinity preferences to their level of education, access to media and health, we will examine the effects of these predictors as well.

## Colombia as our field site

Masculinity preferences have been mostly studied at the population level in developed countries, like the UK or the USA, where people’s access to education, health and media is high. We chose Colombia as it differs substantially from previously studied populations. Colombia, a developing country, is known for being one of the most dangerous places in the world, exhibiting very low indicators of economic growth and development. For example, in Colombia, the homicide rate was 30 times higher than in the UK in 2014. Likewise, life expectancy is 7% shorter for Colombian than British women. If violence has an effect on women’s masculinity preferences, Colombian women’s perceptions of violence increase the likelihood of finding these effects. Additionally, violence indicators in Colombia differ depending on the geographical area. For instance, there is variability in violence perceptions between rural and urban areas. Including urban and rural participants not only increases the variability of violence perceptions but also guarantees that our study is more representative of the population, being mostly non-WEIRD (non-Western, educated, industrialised, rich, democratic). Since online sampling in developing countries (like Colombia) has been found to be unrepresentative (Batres and Perrett 2014), our participants were tested in person.

## Methods

### Participants

The sample consisted of Colombian women living in the two major cities, Bogota and Medellin, and in two surrounding small towns, Cota and La Estrella, respectively. Our total sample was 120 adult (older than 17 years) heterosexual women, with 30 women from each location. Due to the fact that younger women (of reproductive age) are at higher risk of being the targets of violence, we limited our analyses to women younger than 41 years of age. This left a sample of 83 women (mean age ± SD = 26.7 ± 6.01). In order to avoid participants’ bias, blinded methods were used when collecting all data.

### Stimuli used

For the purpose of comparing the effect of own-ethnicity versus other-ethnicity stimuli, three sets of face images were used, one of European descent, one Salvadoran and one Colombian. Salvadoran images were from urban (El Salvador) and rural areas. Colombian images were of participants from urban (Colombia: Bogota and Medellin) and rural areas (Cajica and La Estrella) of Colombia. The European images were taken from an open-access library (3DSK).

The Colombian pictures (40 females, mean age ± SD = 25.4 ± 6.34; and 40 males, mean age ± SD = 24.33 ± 5.22) were included since the study population was all Colombian. The Salvadoran pictures (40 females, mean age ± SD = 25.43 ± 4.64; and 40 males, mean age ± SD = 26.32 ± 5.32, see Batres and Perrett 2014 for details) were included as Colombia and El Salvador are both Hispanic countries. Salvadoran pictures therefore constitute an intermediate level of ethnic similarity to the study population. European pictures (40 females, mean age ± SD = 23.04 ± 3.81; and 40 males, mean age ± SD = 25.25 ± 4.64) were included to be able to compare masculinity preferences for an ethnically distant group.

**Fig. 1:**
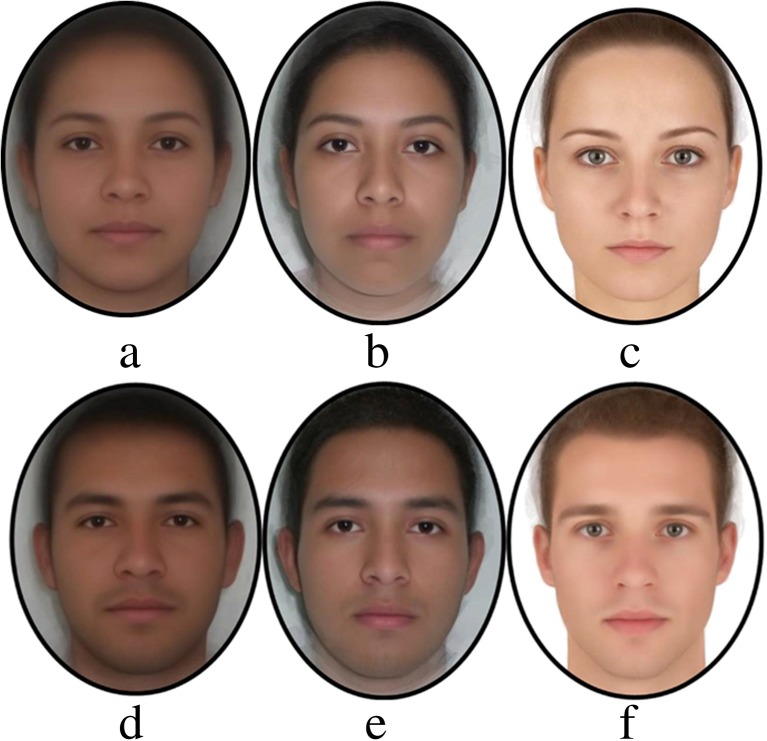
Anchor images (prototypes) for masculinity transforms. Bottom row—women’s facial averages: **a** Colombian, **b** Salvadoran and **c** European. Second row—men’s facial averages: **d** Colombian, **e** Salvadoran and **f** European

**Fig. 2:**
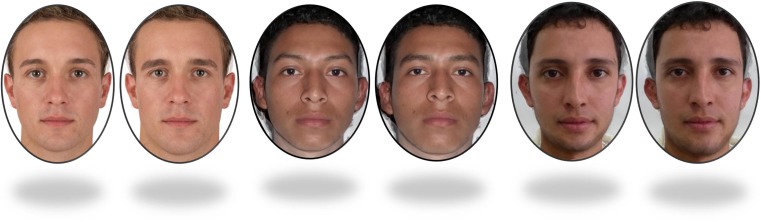
Example pairs of European (left), Salvadoran (middle) and Colombian (right) male facial stimuli. The left of each pair is 50% feminised and the right is 50% masculinised in shape

All images used were taken under constant conditions of light and showed a neutral expression. Using Psychomorph (Version 6), we first delineated each image with 189 points and aligned them to a standard inter-pupillary distance. Three original face images were averaged to make new composites as these would be more representative of the source population than original images of individuals. Five composite images were made for each gender of each ethnicity (Colombian, Salvadoran and European). At the same time, all female and male faces were averaged separately to create gender prototypes for each ethnicity (see Fig. 1). Subsequently, masculinity transforms were made for each composite, by subtracting (or adding) 50% of shape difference between the relevant male and female prototypes (Tiddeman et al. 2001). This resulted in 15 pairs of faces, each consisting of a masculinised and a feminised version of the same face (5 Colombian, 5 Salvadoran and 5 European). See Fig. 2 for an example of each ethnicity (for method details, see Perrett et al. 1998).

Each participant was presented with the 15 pairs of faces one by one in random order but blocked by ethnicity. Each pair was shown in a printed laminated sheet. The left/right position of the masculinised face of the pair and the sheet order between participants were randomised.

Participation in this study consisted of two phases. In the first phase, participants’ masculinity preferences were assessed by showing them the 15 pairs of faces individually and asking which of the two faces (the right or the left) they considered most attractive for each pair (Spanish translation: “Cual de las dos caras le parece mas atractiva?”). The second phase consisted of completing an 82-question survey (questions analysed here are shown in the Appendix). Questions inquired about demographic details (age, gender, number of children, etc.), indicators of health, level of education, access to media and perceptions of violence.

For education level, just one question was asked: “What is your highest level of education?” Participants were given eight options to choose from, ranging from no schooling (illiterate) to graduated from post-graduate studies. To determine the participants’ access to media, three questions were asked: how much time was spent watching national TV, how much time was spent watching cable TV and how frequently they used the internet. Concerning participants’ health, we asked how frequently they were ill during their childhood, how many times on average they had been ill over the past year and how would they rate their health. Violence perceptions were assessed by asking participants how much in danger from violence they felt in the country, in the city/town, and how worried they were when already in bed and realised they left the front door unlocked. Additionally, participants were asked eight questions on how vulnerable to public crime they felt. How much do you worry about falling victim of the following crimes on a regular basis? (1 being not at all–7 all the time)

1. Being attacked by a stranger in the street.
2. Being robbed or mugged in the street.
3. Being harassed, threatened or verbally abused in the street.
4. Being pick-pocketed.
5. Having something stolen in a violent manner.
6. Having your home or property vandalised.
7. Having someone break into your home whilst you or your family are there.
8. Having someone break into your home whilst the inhabitants are away.

An average was computed from the eight questions on vulnerability to public crime. In reference to violence within partnership, seven questions were asked: how likely is a woman/man to be the target of domestic violence in your area?, how vulnerable do men/women feel if they have a confrontation with their partner?, how unsafe do men/women feel if they have a disagreement with their partner about something that really matters to them? and how much do they agree with the statement “men are dangerous to their children”. The three questions relating to domestic violence against women were averaged, and a new variable “domestic violence against women” was created. The same was done for the three questions relating to domestic violence against men to create a new variable “Domestic violence against men”. All questions relating to violence were asked in the abstract “how likely is a woman to be the target of domestic violence in your area”, rather than personalising questions “how likely are you to be the target of domestic violence”. This was done to avoid disclosure. All participants were debriefed and given contact details for the local police and priest in case they needed to make personal reports.

#### Data availability

All data generated or analysed during this study are included in this published article as supplementary material (Data_Behavioral_Ecology_submission_Colombia_data_set_3.sav).

## Variables analysed

### Dependent variable

Participants’ masculinity preferences were calculated as the percentage of faces high in masculinity that were selected as more attractive across the pairs. As we had three ethnicities in our stimuli, we calculated a percentage masculinity preference for each stimulus ethnicity: Colombian, European and Salvadoran.

### Independent variables

#### Factor analysis

When possible, factors were extracted from the violence, education, development and health questions via principal component analysis. The factorability of the questions relating to each indicator was evaluated via the Kaiser-Meyer-Olkin measure of adequacy (when greater or equal to 0.5, it was accepted) and Bartlett’s test of sphericity (accepted when *p* ≤ 0.05). All solutions were un-rotated and based on eigenvalues greater than 1. All loadings are shown in Tables 1, 2 and 3. Scores were saved as new variables/factors.

**Table 1:** Correlation matrix for factors extracted from the questions related to violence (bold indicates variables that have a greater than 0.4 correlation value)

Indicators	Factor and loadings	
	*Public violence*	*Domestic violence*
Average violence against women	0.27	**0.67**
Average violence against men	− 0.06	**0.86**
Men dangerous to children	0.42	**0.56**
Average vulnerability	**0.60**	0.03
Danger city/town	**0.67**	− 0.19
Danger country	**0.65**	− 0.35
Locking door	**0.61**	0.43

**Table 2:** Correlation matrix for factors extracted from the questions related to health

Indicators	Factor and loadings
	*Illnesses*
Health rating	− 0.76
Average illnesses reported for the last year	0.47
Frequency of illnesses during childhood	0.76

**Table 3:** Correlation matrix for factor analysis for questions related to TV access

Indicators	Factor and loadings
	*TV*
Time spent watching national TV	0.87
Time spent watching cable TV	0.87

#### Violence factors

A total of 17 questions were asked in reference to perceived violence. These were reduced to seven variables once the answers to questions on vulnerability to public crime, domestic violence against women and domestic violence against men were separately averaged. An exploratory factor analysis of the seven variables revealed two factors. The first factor loaded mainly on questions related to public violence and the second one loaded mostly on questions relating to violence within partnership and against children (see bold correlations in Table 1). The first factor is referred to as *public violence* and the second factor as *domestic violence*. Respectively, these factors explained 26.5 and 23.9% of the variance from the violence questions.

#### Health factors

Participants’ ratings of their health, the frequency of illnesses during the last year and during childhood were included in this factor analysis. One factor was extracted (shown in Table 2), explaining 45.9% of the variance in the answers to these questions. Inclusion of other questions did not meet the assumptions of the Kayser-Meyer-Olkin sample adequacy principle.

### Media access

Only questions related to time spent watching national and cable TV were included in the factor analysis to meet the Kayser-Meyer-Olking measure of sampling adequacy. One factor was extracted, explaining 74% of the variance in answers to these questions (Table 3). Internet frequency use was included in the analysis as an additional binary variable with low- and high-use categories.

### Education level

The highest level of education was introduced in subsequent analyses on its own.

## Results

A repeated-measures ANOVA was run, with masculinity preferences for male faces as the dependent variable, face ethnicity (i.e. European, Salvadoran or Colombian) as the within-subject factor and having children or not as a between-subjects factor. Along with age, the factors/variables described in the previous section (i.e. domestic violence, public violence, illnesses, time spent watching TV, internet frequency and education level) were included as covariates in this model. Residuals from this model (residual masculinity preferences for European, Salvadoran and Colombian male faces) were all normally distributed (all *p* > 0.099).

**Fig. 3:**
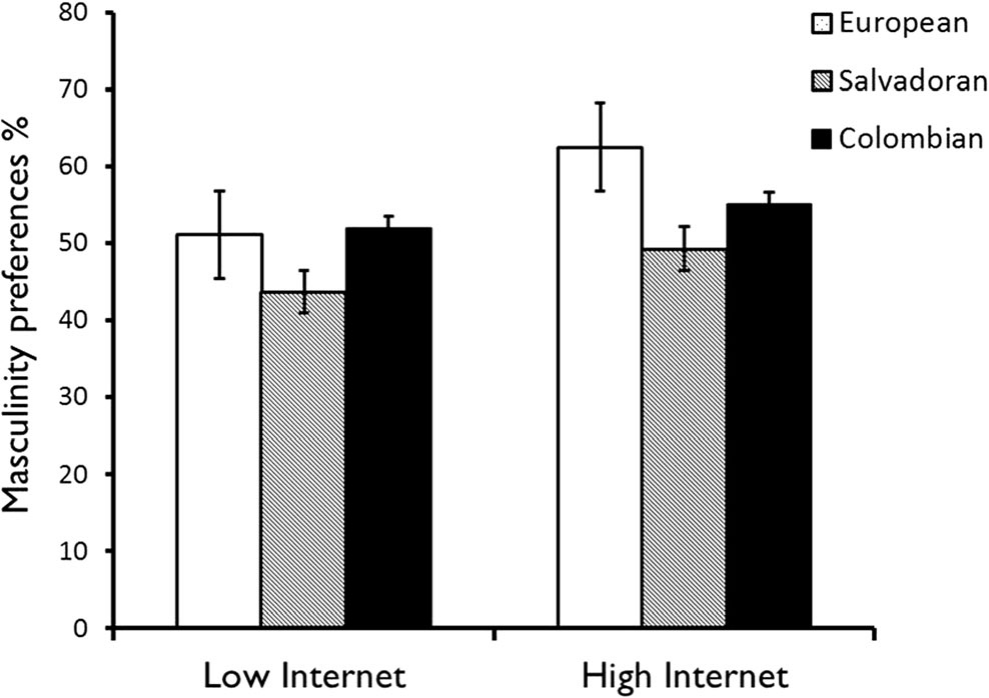
Effect of internet frequency use on masculinity preferences (%) for male faces of different ethnicities. Standard error bars are shown

The interaction between ethnicity of the stimuli used and internet frequency was marginally significant, F(2,83) = 3.001, *p* = 0.052, suggesting that masculinity preferences for the three types of male faces differed depending on the amount of internet used (Fig. 3). Participants’ masculinity preferences for Colombian male faces were unaffected by internet frequency (mean masculinity preference percentage for high internet frequency *M* = 55.0 and for low internet frequency *M* = 52.0), whereas masculinity preferences for European and Salvadoran faces were higher with more frequent internet use (European: high internet frequency *M* = 62.5 and low internet frequency *M* = 51.1; Salvadoran: high internet frequency *M* = 43.7 and low internet frequency *M* = 49.3).

Face stimulus ethnicity did not interact with any other variable (i.e. age, having children, time spent watching TV, education level, public violence and illnesses factors; all *p* > 0.075). Additionally, none of the variables/factors had a significant main effect on masculinity preferences (all *p* > 0.19). Due to the marginally significant interaction described previously, subsequent univariate analyses were done separately for each face ethnicity.

### Masculinity preferences for Colombian male faces

**Fig. 4:**
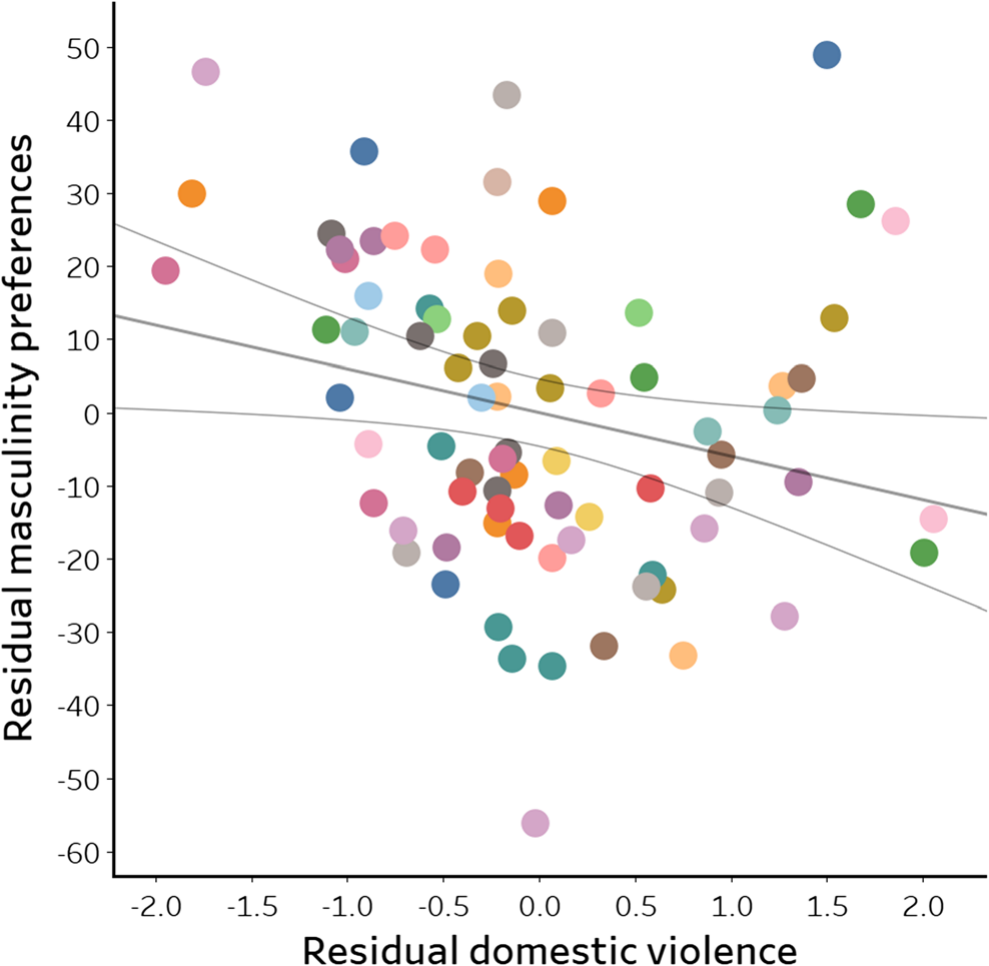
Effect of domestic violence on women’s preferences for masculinity in Colombian male faces. Lines above show the least squares trend line and 95% confidence intervals. Different colours indicate each participant. (Residual domestic violence plotted against the unstandardised residuals of masculinity preference from the univariate model, controlling for age, children, health, TV access, internet and public violence)

Domestic violence had a significant effect on women’s masculinity preferences for Colombian male faces (F(1,78) = 4.57, *p* = 0.036, *β* = − 5.96, *η* ^2^ = 0.062). When women had higher perceptions of risk for domestic violence in their surroundings, their masculinity preferences were lower (Fig. 4). No other factor/variable significantly predicted women’s masculinity preferences (all *p* > 0.11).

### Masculinity preferences for Salvadoran or European male faces

Preferences for masculine male faces from El Salvador were not affected either by the domestic violence factor (F(1,78) = 0.16, *p* = 0.69, *β* = − 1.2, *η* ^2^ = 0.002) or any other variable/factor, all *p* > 0.11. Likewise, masculinity preferences for European faces were not affected either by domestic violence (F(1,78) = 0.001, *p* = 0.98, *β* = 0.106, *η* ^2^ = 0.000) or any other variable/factor (i.e. having children, age, public violence, illnesses, time spent watching TV, internet frequency and education level; all *p* > 0.80).

## Discussion

Our findings are in line with some of our predictions. First, we reasoned that worries about violence might differentiate into two types of concern: that for public violence and that for domestic violence. This separation was apparent in the factor analysis of violence concerns. The results presented here indicate that women’s masculinity preferences for male faces are influenced by their perceptions of danger from violence, and that this influence depends on the source of violence. We did not find support for our prediction that concerns about public violence would be linked to a greater masculinity preference. There was support for the prediction about domestic violence. When women thought there was a higher likelihood of domestic violence in their surroundings, they showed lower masculinity preferences for male faces. Women’s masculinity preferences for male faces were also contingent on the ethnicity of the face stimuli shown to participants. There was a significantly lower masculinity preference for Colombian male faces when women felt there was a higher risk of domestic violence. Even after controlling for participant’s age, having children, illnesses, education, internet access frequency and time spent watching TV, the effects of domestic violence remained significant. Moreover, domestic violence was the only variable that contributed significantly to explaining the variation in women’s masculinity preferences.

### Violence as a multi-modal factor

Most studies testing the effects of violence on mate preferences, both at the population (Brooks et al. 2010) and at the individual level (Snyder et al. 2011; Li et al. 2014; Borras-Guevara et al. 2017), have studied violence as a uni-modal factor. It is true that when one domain of violence increases, other domains may show the same pattern. For instance, public violence in North India correlated positively with elevated risks of domestic violence against women (Koenig et al. 2006). It should be noted that different violence domains have different actors as perpetrators and as victims. Public violence is known to be mostly driven by violence between men. This has been shown extensively through Daly’s work on the “Young male syndrome”. Gang violence in Detroit was found to be overrepresented by young men for example (Wilson and Daly 1985). By contrast, in most cases of domestic violence, men act as the perpetrators and women are the victims (Zlotnick et al. 1998; Brewster et al. 2002; Miller 2005). This is evident from studies in the USA, where only 16% of domestic violence disputes were perpetrated by women (Miller 2005). Reciprocally, 73% of injury reports from within-partnership violence were from women (Zlotnick et al. 1998). Accordingly, women should show different concerns about domestic violence and public violence. The results from our factor analysis corroborate that violence is not a uni-modal factor and can arise independently, either from within the household (domestic violence) or outside the household (public violence). It is notable that concerns about men’s violence against children, women’s violence against men and men’s violence against women all group together (Table 1). This is consistent with the co-occurrence of multiple forms of domestic violence (Coulter and Mercado-Crespo 2015).

To understand the trade-off women face in partner selection, one needs to define the perceived frequency and severity of the different forms of violence. When domestic violence is perceived to be rare but public violence is common, protection may become a priority. In the field, future investigations could ask women what type of violence they fear more, violence from a stranger (public violence) or violence from a partner (domestic violence). Additionally, in the laboratory, participants could be primed with different scenarios where public violence was high but domestic violence was low, and vice versa. Equating the nature of aggression in the two scenarios might allow the relative impact that public and domestic violence have on mate choice preferences to be compared. The dangers from public and domestic violence could also impact on preferences for different mate characteristics (e.g. formidability and intention; Lustgraaf et al. 2015).

### The influence of violence on women’s masculinity preferences

Being more aggressive, stronger, dominant and more likely to cheat on a partner are just some of the negative traits that are associated with men’s masculinity (Booth and Dabbs 1993; Boothroyd et al. 2007; Fink et al. 2007; Jones et al. 2010). These associations may explain why the women studied here prefer less masculine male partners when they feel there is a higher risk of domestic violence. This preference may be a reflection of women’s strategy to avoid those partners who are more likely to behave aggressively and dangerously towards women or their offspring. Consistent with this explanation, living in environments where within-partnership violence is common would be a scenario where it would be advantageous to prefer less masculine partners. In support of this reasoning, Li et al. (2014) found that when women were shown images of males hitting females, their preferences for masculine male faces were disrupted. Additionally, when Colombian women agreed more with men being dangerous to their children, their masculinity preferences for Salvadoran male faces were lower (Borras-Guevara et al. 2017). Furthermore, in comparison to men, women invest a lot more energy and time in their offspring (Trivers 1972; Geary 2000). This makes it especially important for women to be able to recognise facial cues in potential partners that hint at aggressive tendencies and untrustworthiness (Stirrat and Perrett 2010).

### Effects of violence on masculinity preferences depend on the ethnicity of facial stimuli

When women thought there was a higher likelihood of domestic violence in their surroundings, their masculinity preferences for Colombian male faces were significantly lower. Since participants in this study are more likely to form a partnership with a Colombian male, these results may indicate that women are more sensitive to masculinity cues and their associations in Colombian faces.

### Masculinity preferences at the individual level

An increase in women’s preferences for masculinity in partners has been claimed to be a result of their need for protection in harsh/violent settings (Brooks et al. 2010; Puts 2010; Snyder et al. 2011; Ryder et al. 2016). More recent studies, however, have suggested that a reduction in women’s masculinity preferences may reflect an additional strategy to avoid men who are more likely to be aggressive within the home (Borras-Guevara et al. 2017). Our results support this latter claim; as in the present study, women who thought there was a higher likelihood of domestic violence preferred less masculine male faces. On the other hand, at the population level, women from countries with high-income inequality, which is associated with high public violence, showed higher masculinity preferences than women from countries with low-income inequality (Brooks et al. 2010; but see Debruine et al. 2011). The results presented here show that public violence did not influence women’s masculinity preferences, whereas domestic violence did. The divergence of the results presented here and the interpretation of Brooks et al. (2010) may reflect that when Colombian women are faced with risks related to within-partnership violence and/or public violence, the former will matter more in partner preferences. Additionally, the fact that traditional social roles are still the norm in Colombia, men being the providers and women the housekeepers, could also explain why women worry more about domestic violence as they spend most of their time at home where risks of being hurt would most likely come from partners. Furthermore, our results are at the individual level, whereas Brooks’ results are based on consideration of country-level indicators. Aggregating measures at the population level could conflate the effects of multiple independent factors. For example, indexes of pathogen load and homicide rates could be correlated with each other at the population level yet have independent effects on masculinity preferences at the individual level. This type of discrepancy between population and individual levels of analyses has been found before (see Pollet et al. 2014). Since Snyder et al. (2011) found that women’s formidability preferences were explained by individual vulnerability to crime but not by neighbourhood crime, it seems that large-scale measures may not indicate what is happening at the individual level.

Vulnerability to public violence predicted women’s preferences for more formidable male bodies as well as male personality characteristics associated with formidability (Snyder et al. 2011). Results presented here do not challenge those of Snyder’s; we believe that women’s preferences for masculine partners depend on the extent of the risks associated with violence, both from strangers and from within the partnership. If women or their partners are more likely to experience public violence than domestic violence, then it would be in their interest to prefer male partners or friends who can offer better protection (i.e. men with masculine formidable characteristics). On the other hand, if the risks of domestic violence are higher than those of public violence, then women may prefer partners who are less likely to be aggressive (i.e. men who are less masculine and formidable), as shown in our results.

## General conclusion

In accordance with the trade-off theory (Gangestad and Simpson 2000; Thornhill and Gangestad 2006), our results show the advantages of low masculinity within the home environment. We found a negative relationship between women’s masculinity preferences for male faces and likelihood of domestic violence: Colombian women who thought there was a higher likelihood of domestic violence had lower masculinity preferences for Colombian male faces. Whereas former studies have mostly focused on the influence of environmental factors at the population level, the findings presented here indicate that individual perceptions of domestic violence affect women’s masculinity preferences for male faces.

### Electronic supplementary material

ESM 1 (XLSX 33 kb)

## Appendix

### Questionnaire

#### English version

1. **Demography**
2. How old are you?
3. Please indicate your gender at birth.
  - Female
  - Male
  - Other
  - Prefer not to say
    i. If female, at what age did you get your first period?
4. Please indicate how would you rate your sexual orientation? (1 completely homosexual - 4 bisexual - 7 completely heterosexual). You can skip this question if you would prefer.
5. Are you currently in a committed relationship? (Yes/No)
  - If not, would you like to be in a committed relationship? (Yes/No)
6. Do you have any children? (Yes/No)
  - If yes, how many children do you have?
  - If yes, please indicate what is the age of your youngest child: ____
7. **Health**
8. In general, how would you rate your health? (1 poor - 7 excellent)
9. On average how many times a year do you get sick, so that you are confined to stay in bed?
10. During childhood (before 13 years old) how often in a year did you get a serious illness that confined you to bed? (1 never - 7 more than 5 times a year)
11. **Education**
12. Please indicate the highest level of education you attained or are currently enrolled in.

- illiterate/ attended primary school/ completed primary school/ attended secondary school/ completed secondary school/ attended university/ completed undergraduate degree/ attended postgraduate degree/ completed postgraduate degree

4. **Development**
1. How many times a week do you have access to the internet?
  - 0 - 2
  - 2 - 4
  - 4 - 6
  - All the time
2. Do you have TV at home? (Yes/No)
  - If you answered yes, how much national TV you watch per day?
    i. 30 minutes
    ii. 1 hour
    iii. 2 hours
    iv. 3 hours
    v. More than 3 hours
3. Do you have foreign/cable TV at home?
  - If you answered yes, how much foreign TV you watch per day?
    i. 30 minutes
    ii. 1 hour
    iii. 2 hours
    iv. 3 hours
    v. More than 3 hours
5. **Fear of violence**
1. In general, how much in danger from violence do you feel in the following places? (1 “feel no danger at all” - 7 “feel very much in danger”).

- In town/city
- In the country

2. From 1 to 7, how worried do you feel when you are already in bed and you realise you have forgotten to lock/block your home front door? (1 “not at all worried, I would sleep like a baby” - 7 being “very worried, I would jump out of bed straight away to lock/block the door”).
6. **Perceived vulnerability to public violence**

How much do you worry about falling victim of the following crimes on a regular basis? (1 being not at all - 7 all the time)

1. Being attacked by a stranger in the street
2. Being robbed or mugged in the street
3. Being harassed, threatened or verbally abused in the street
4. Being pick-pocketed
5. Having something stolen in a violent manner
6. Having your home or property vandalised
7. Having someone break into your home whilst you or your family are there
8. Having someone break into your home whilst the inhabitants are away
9. **Domestic violence**
10. How likely is a woman to be the target of domestic/partner violence in your area (e.g. neighbourhood, town)? (1 not likely at all - 7 very likely)
11. How likely is a guy to be the target of domestic/partner violence in your area (e.g. neighbourhood, town)? (1 not likely at all - 7 very likely)
12. How much do you agree with the following statement “Men are dangerous to their own children” (1 completely disagree - 7 completely agree).
13. **Vulnerability within own relationship**
14. How vulnerable do women (in your area/town) feel if they have a confrontation with their partner? (1 not vulnerable at all - 7 very vulnerable).
15. If a woman (in your town) disagrees with her partner about something that really matters to her, is she likely to feel safe enough to tell him (1 very likely to feel safe enough - 7 not very likely to feel safe enough).
16. How vulnerable do men (in your area/town) feel if they have a confrontation with their partner? (1 not vulnerable at all - 7 very vulnerable).
17. If a man (in your town) disagrees with his partner about something that really matters to him, is he likely to feel safe enough to tell her (1 very likely to feel safe enough - 7 not very likely to feel safe enough).

#### Spanish version

1. **Demografia**
  1. Cuál es su edad?
  2. Por favor indique su sexo al momento de su nacimiento?

- Hombre
- Mujer
- Otro
- Prefiere no responder

i. Si respondio que es mujer, a que edad le llego su primera menstrucaion?
  3. Por favor indique como clasificaria su orientacion sexual? (1 completamente homosexual-4 bisexual y 7 completamente heterosexual).
  4. Esta usted en una relacion?
  5. Tiene hijos?

- Si respondio si, cuantos hijos tiene?
- Si respondio si, cual es la edad de su hijo menor?

2. **Salud**
  1. En general como clasificaria su salud? (1 mala - 7 excelente)
  2. En promedio, cuantas veces al año se enferma usted?
  3. Durante su niñez (antes de los 13 años) que tan frecuentemente una enfermedad grave lo obligo a estar en cama? (1 nunca - 7 mas de 5 veces al ano)
3. **Educacion**
  1. Por favor indique su nivel mas alto de estudios?

- analafabeta/ atendio la primaria/ se gradua de la primaria/ atendio el bachillerato/ se gradua del bachillerato/ atendio la universidad/ se graduo de la universidad/atendio el post-grado/se gradua del post-grado.

4. **Desarrollo**
  1. Cuantas veces a la semana tiene usted acceso al internet?

- 0 - 2
- 2 - 4
- 4 - 6
- Todo el tiempo
  2. Tiene usted television en casa?
- Si respondio si, cuanta television nacional ve usted al dia?
  i. 30 minutos
  ii. 1 hora
  iii. 2 horas
  iv. 3 horas
  v. Mas de 3 horas
  3. Tiene usted television por cable/parabolica en casa?
- Si respondio si, cuanta television por cable/parabolica ve usted al dia?
  i. 30 minutos
  ii. 1 hora
  iii. 2 horas
  iv. 3 horas
  v. Mas de 3 horas

5. **Violencia**
  1. En general que tan seguro frente a la violencia se siente usted en los siguientes lugares? En una escala de 1 a 7, 1 siendo ningún riesgo a su seguridad y 7 alto riesgo a su seguridad.

- En la ciudad/pueblo
- En su país
  2. Usted se encuentra listo para dormir, ya entre su cama, de 1 a 7, que tanto le preocuparia a usted dejar la puerta de su casa abierta? (1 no me preocuparía en lo absoluto, dormiria como un bebe - 7 me preocuparía demasiado, saltaria de la cama a cerrar la puerta).

6. **Vulnerabilidad a violencia publica**

Que. tanto se preocupa usted regularmente de ser victima de los siguientes crímenes (en una escala de 1 a 7, 1 no se preocupa en lo absoluto - 7 se preocupa todo el tiempo).

1. Ser atacado por un extraño en la calle
2. Ser robado en la calle
3. Ser acosado, amenazado o abusado verbalmente en la calle
4. Ser chalequiado en la calle
5. Que. alguien le robe de manera violenta
6. Que. roben o vandalicen su casa
7. Que. alguien entre a robar a su casa cuando esta esta sola
8. Que. alguien entre a robar su casa cuando hay gente allí
9. **Violencia domestica**
  1. Que. tan probable es que una mujer sea objeto de violencia domestica en su barrio? (1 no es para nada probable - 7 muy probable).
  2. Que. tan probable es que un hombre sea objeto de violencia domestica en su barrio? (1 no es para nada probable - 7 muy probable).
  3. Que. tan de acuerdo esta usted con la siguiente afirmación? “Los hombres pueden ser un peligro para sus propios hijos”
10. **Vulnerabilidad en pareja**
  1. Que. tan vulnerables se sientes las mujeres en su barrio si tienen una confrontación con su pareja? (1 no se sienten vulnerables - 7 muy vulnerables)
  2. Si una mujer esta en desacuerdo con su pareja con respecto a algo que realmente le importa a ella, que tan probable es que ella se sienta a salvo de comunicárselo a su pareja? (1 muy probable que se sienta a salvo - 7 poco probable que se sienta a salvo)
  3. Que. tan vulnerables se sientes los hombres en su barrio si tienen una confrontación con su pareja? (1 no se sienten vulnerables - 7 muy vulnerables)
  4. Si un hombre esta en desacuerdo con su pareja con respecto a algo que realmente le importa a el, que tan probable es que el se sienta a salvo de comunicárselo a su pareja? (1 muy probable que se sienta a salvo - 7 poco probable que se sienta a salvo)
